# Legume Plant Peptides as Sources of Novel Antimicrobial Molecules Against Human Pathogens

**DOI:** 10.3389/fmolb.2022.870460

**Published:** 2022-06-09

**Authors:** Rui M. Lima, Balaji Baburao Rathod, Hilda Tiricz, Dian H. O. Howan, Mohamad Anas Al Bouni, Sándor Jenei, Edit Tímár, Gabriella Endre, Gábor K. Tóth, Éva Kondorosi

**Affiliations:** ^1^ Institute of Plant Biology, Biological Research Centre, ELKH, Szeged, Hungary; ^2^ Department of Medical Chemistry, Albert Szent-Györgyi Medical School, University of Szeged, Szeged, Hungary

**Keywords:** legume NCR peptides, antimicrobial activity, ESKAPE pathogens, *Acinetobacter baumannii*, *Candida albicans*, mode of action

## Abstract

Antimicrobial peptides are prominent components of the plant immune system acting against a wide variety of pathogens. Legume plants from the inverted repeat lacking clade (IRLC) have evolved a unique gene family encoding nodule-specific cysteine-rich NCR peptides acting in the symbiotic cells of root nodules, where they convert their bacterial endosymbionts into non-cultivable, polyploid nitrogen-fixing cells. NCRs are usually 30–50 amino acids long peptides having a characteristic pattern of 4 or 6 cysteines and highly divergent amino acid composition. While the function of NCRs is largely unknown, antimicrobial activity has been demonstrated for a few cationic *Medicago truncatula* NCR peptides against bacterial and fungal pathogens. The advantages of these plant peptides are their broad antimicrobial spectrum, fast killing modes of actions, multiple bacterial targets, and low propensity to develop resistance to them and no or low cytotoxicity to human cells. In the IRLC legumes, the number of NCR genes varies from a few to several hundred and it is possible that altogether hundreds of thousands of different NCR peptides exist. Due to the need for new antimicrobial agents, we investigated the antimicrobial potential of 104 synthetic NCR peptides from *M. truncatula, M. sativa, Pisum sativum, Galega orientalis* and *Cicer arietinum* against eight human pathogens, including ESKAPE bacteria. 50 NCRs showed antimicrobial activity with differences in the antimicrobial spectrum and effectivity. The most active peptides eliminated bacteria at concentrations from 0.8 to 3.1 μM. High isoelectric point and positive net charge were important but not the only determinants of their antimicrobial activity. Testing the activity of shorter peptide derivatives against *Acinetobacter baumannii* and *Candida albicans* led to identification of regions responsible for the antimicrobial activity and provided insight into their potential modes of action. This work provides highly potent lead molecules without hemolytic activity on human blood cells for novel antimicrobial drugs to fight against pathogens*.*

## Introduction

The WHO priority pathogens, known also as ESKAPE bacteria, are *A. baumannii, Pseudomonas aeruginosa*, *Klebsiell* *a pneumoniae, Escherichia coli* and *Enterobacter* spp. at the highest priority, followed by *Enterococcus faecium, Staphylococcus aureus*, *Helicobacter pylori, Salmonella* spp.*, Campylobacter* and *Neisseria gonorrhoeae*, which are prone to escape from conventional antibiotics and treatment (Bhatia et al., 2021). *A. baumannii* infections are rising at a faster rate than other ESKAPE bacteria, making carbapenem-resistant *A. baumannii* the number one target for antibiotic development (Harding et al., 2018; Kyriakidis et al., 2021). The WHO also lists various *Candida* species as prominent fungal pathogens (Oliveira et al., 2021), since invasive *Candida* infections cause hundreds of thousands of deaths each year. Multidrug-resistant strains are rapidly appearing and spreading around the world, threatening to render our antifungal arsenal unusable. Hospital-acquired infections have made everyday surgeries such as cesarean sections, hip replacements, cancer treatments and organ transplants more dangerous (Fernando et al., 2017). In the current COVID-19 pandemic, hospitalized patients are at increased risk of respiratory bacterial coinfection caused by *Acinetobacter* (Rangel et al., 2021) and of severe *Candida* infections because of their impaired immune systems (Moser et al., 2021).

To be effective against these drug-resistant bacteria and fungi, new antimicrobials are required with novel modes of action that have little or no toxicity for human cells. Antimicrobial peptides (AMP), which are host defense agents (Zasloff, 2002; Egorov et al., 2005; Campos et al., 2018; Li et al., 2021), are promising candidates for novel types of effective antimicrobials based on their broad antimicrobial spectrum, different mechanisms of action, and low tendency for resistance development (Magana et al., 2020). The weaknesses of certain natural AMPs can be their lower stability and incidental toxicity, which can be overcome with designed modifications of synthetic peptides (Büyükkiraz and Kesmen, 2021).

AMPs have a wide variety of molecular configurations, but the majority are linear peptides found in plants, insects and mammals. The amphiphilic nature of peptides and the presence of positively charged residues enable their attachment and insertion into the bacterial membrane, resulting in membrane permeabilization and their antibacterial action (Hancock, 1997; Yamaguchi and Huffaker, 2011). The antifungal effect of AMPs is mostly attributed to permeabilization and/or the formation of holes in the fungal membranes (Cesare et al., 2020).

Most known AMPs are of animal origin (Wang et al., 2016), however, plants have evolved an extremely rich source of AMPs that act as the most prominent defense barriers. The largest group of plant AMPs are cysteine-rich defensins characterized by four conserved disulfide bridges, positive net charge and amphipathic character and capability to interact with microbial membranes, and by entering the cells to inhibit translation and enzymatic activity (Maróti et al., 2015; Tam et al., 2015).

In the Inverted Repeat Lacking Clade (IRLC) of legumes, in addition to defensins, a related family of peptides has evolved that encodes nodule specific, cysteine-rich NCR peptides that play essential roles in symbiosis (Mergaert et al., 2003; Nallu et al., 2013; Montiel et al., 2017). Legume plants in symbiosis with their rhizobium bacterium partner form nitrogen-fixing root nodules. The primary role of the NCR peptides is to convert bacteria into large polyploid, non-cultivable nitrogen-fixing bacteroids in the nodule cells (Van de Velde et al., 2010). This is a terminal differentiation process as bacteroids permanently lose their cell division potential, therefore some of the peptides must be involved in inhibiting the growth of bacteria and may exert antibacterial activity *in vitro*. In *M. truncatula* ∼700 genes code for secreted NCR peptides that are targeted to the bacteroids via the secretory pathway. Mature MtNCR peptides range in size from 24 to 65 amino acids, but most of them composed of 30–50 amino acids and contain 4 or 6 cysteine residues at conserved positions in a highly variable amino acid sequence (Mergaert et al., 2003; Mergaert, 2018; Lima et al., 2020; Roy et al., 2020). Until now, only a few NCRs have been tested for antimicrobial activity against human, animal and plant pathogenic bacterial and fungal strains (Tiricz et al., 2013; Balogh et al., 2014; Ördögh et al., 2014; Mikuláss et al., 2016; Farkas et al., 2017; Montiel et al., 2017; Velivelli et al., 2020; Isozumi et al., 2021; Szerencsés et al., 2021). In this respect, the most studied NCR is the highly cationic NCR247 (pI: 10.15) and its derivatives, which are highly effective against ESKAPE pathogens, in most cases outperforming levofloxacin, a third-generation fluoroquinolone antibiotic under laboratory conditions (Jenei et al., 2020). The regions responsible for the antimicrobial activity of NCRs were delineated by synthesizing their shorter and substituted derivatives, which in some cases even had increased activity (Jenei et al., 2020; Isozumi et al., 2021; Szerencsés et al., 2021). The reduced forms of NCR247 and NCR335 were found to be more effective *in vitro* against the natural symbiont *Sinorhizobium meliloti* Rm2011 than the oxidized variants forming the disulfide bridges (Ribeiro et al., 2017). The reduced NCR247 was also more effective against *E. coli* than any of the three disulfide regioisomers and activity was retained even when all four cysteines in NCR247 were replaced with serine (Shabab et al., 2016).

IRLC includes 56 genera and more than 4,000 species (Duan et al., 2021), and presumably all species have NCRs as it was confirmed in a few selected species. The genus *Astragalus,* with 2,300–2,500 members, has the highest number of species among angiosperms (Duan et al., 2021). In *A. canadensis* 108 NCRs have been identified (Montiel et al., 2017), while in the members of the Vicioid subclade 200–700 NCRs (Ištvánek et al., 2014; Montiel et al., 2017), thus, it is reasonable to assume that IRLC species in total express hundreds of thousand different NCRs, providing almost unlimited amount of bioactive peptides and likely many AMPs. However, not all NCRs have antimicrobial activity as it would not be compatible with the symbiotic functions and maintaining the endosymbionts alive. Antimicrobial activity of MtNCRs was reported for cationic peptides with positive net charge (NC) except for the anionic NCR211 with −2.1 net charge (Kim et al., 2015).

In this work we tested the antimicrobial activity of 104 chemically synthetized NCR peptides; 78 NCRs from *M. truncatula* and 26 peptides from other IRLC legumes; from *P. sativum, G. orientalis, C. arietinum* and *M. sativa* against ESKAPE pathogens. From the latter group, shorter peptide derivatives were also synthesized and tested against *A. baumannii* and *C. albicans*.

## Materials and Methods

### Chemical Synthesis of Peptides

The NCR peptides and their derivatives (Table 1 and Supplementary Table S1) were chemically synthesized with–COOH or–CONH_2_ C-terminus. Peptides with–COOH -terminus were purchased from ProteoGenix (France), GenicBio Limited (People’s Republic of China), BBI Life Sciences Corporation (People’s Republic of China). Peptides with C-terminal–CONH_2_ group were synthesized using an automated peptide synthesizer (CEM Liberty Blue) and TentaGel S RAM resin (loading of amino groups 0.23 mmol/g) according to the standard protocol of solid-phase peptide synthesis (SPPS) and as described before (Jenei et al., 2020). The isoelectric point (pI) and net charge (NC) of the peptides were calculated with https://pepcalc.com/. The calculation of other physicochemical properties were done with DBAASP v3 (Pirtskhalava et al., 2021) and AMP predictions were carried out with CS-AMPPred (Porto et al., 2012), CAMP_R3_ (Waghu et al., 2016; Waghu and Idicula-Thomas, 2020), Antimicrobial Peptide Scanner vr.2 (Veltri et al., 2018), DBAASP v3 (Pirtskhalava et al., 2021) and Sense the moment (Porto et al., 2022).

**TABLE 1:** Antimicrobial activities of NCR peptides against *A. baumannii* and *C. albicans*.

Name	Amino acid sequence	pI	NC	MBC	MFC
CaNCR13	KPCQSDKDCKKFACRKPKVPKCINGFCKCVRIW-COOH	**9.93**	**7.60**	**6.25**	**6.25**
CaNCR63	KMICKTRVDCKKYRCPRSKIKDCVKGYCRCVRKK-COOH	**10.48**	**11.60**	**6.25**	**3.125**
CaNCR63_1-20_	KMICKTRVDCKKYRCPRSKI-CONH_2_	**10.85**	**7.80**	**6.25**	**25**
CaNCR63_15-34_	CPRSKIKDCVKGYCRCVRKK-CONH_2_	**10.65**	**7.70**	**6.25**	**3.125**
MsNCR443	ESIECRTVADCPKLISSKFVIKCIKKRCVAQFFK-COOH	**9.68**	**4.70**	**6.25**	**6.25**
MsNCR463_16-35_	CKPKRGVNFRCRKGKCFPVR-CONH_2_	**11.79**	**8.80**	**1.6**	**3.125**
MsNCR463_17-30_	KPKRGVNFRCRKGK-CONH_2_	**12.45**	**7.90**	**3.125**	**3.125**
PsNCR349	YNLKYCTNDKDCPTMMCFPPDVSKCVWKTCYCVQKHKKKLKKKKKLTFNM-COOH	**9.93**	**9.70**	**3.125**	**3.125**
PsNCR349_26-50_	VWKTCYCVQKHKKKLKKKKKLTFNM-CONH_2_	**11.06**	**11.00**	**3.125**	**3.125**
PsNCR349_31-50_	YCVQKHKKKLKKKKKLTFNM-CONH_2_	**11.24**	**10.00**	**6.25**	**1.6**
PsNCR352	PSGLRCLNDSDCLRFRCSKIYKVLCIERRCRRIKMH-COOH	**10.30**	**6.80**	**6.25**	**3.125**
Ampicillin				**10240**	
Miconazole					**50580**

pI, isoelectric point; NC, net charge; MBC, minimal bactericidal concentrations of the peptides and ampicillin against *A. baumannii* in μM; MFC, minimal fungicidal concentrations of the peptides and miconazole against *C. albicans* in μM.

### Antimicrobial Activity Assay

The following pathogenic bacterial strains were used: The Gram-positive *Enterococcus faecalis* (ATCC 29212), *Staphylococcus aureus* (HNCMO112011), *Listeria monocytogenes* (ATCC 19111) and the Gram-negative *Pseudomonas aeruginosa* (ATCC 27853), *Escherichia coli* (ATCC 8739), *Salmonella enterica* (ATCC 13076), *Klebsiella pneumoniae* (NCTC 13440), *Acinetobacter baumannii* (ATCC 17978) obtained from the ATCC (United States) and NCTC (National Collection of Type Cultures–England). For the antifungal experiment the *Candida albicans* W01 strain (Ördögh et al., 2014) was used. Antimicrobial activities of peptides against ∼10^7^ log phase bacteria were tested in Potassium-Phosphate Buffer (PPB, pH 7.4) as described by Jenei et al. (2020) while anti-*Candida* assays were done according to Szerencsés et al. (2021). The two-fold dilution series of peptides ranged from 25 μM to 0.125 μM, while that of the antibiotics ampicillin (Merck) and miconazole (Duchefa Biochemie) from 10.24 mM to 0.1 μM. The lowest concentration of the antimicrobial agents, which completely eliminated viable bacteria or *C. albicans* were considered as the minimal bactericidal concentration (MBC) and minimal fungicidal concentration (MFC).

### Biofilm Degradation Assay

To test the possible effect of NCRs on the already formed biofilms, *A. baumannii* and *C. albicans* (at starting OD_600_ = 0.1) were first incubated in 96 well plates containing 200 μl LB broth or YPD broth, respectively at 37°C for 48 h to allow biofilm formation (Kanugala et al., 2019). After formation of the biofilm 160 µl were gently discarded from the wells and then NCR solutions were added at 25, 50 and 100 µM final concentrations in the volume of 200 µl for overnight treatment (Montiel et al., 2017). Then, the unattached suspending cells were discarded and the remaining biofilms were washed twice with PBS. The bound biofilms were stained with 35 μl of 0.1% methanolic crystal violet for an hour at room temperature. Excess crystal violet was removed by washing the wells twice with distilled water. The stained biofilms were dried overnight at room temperature and then solubilized with 200 µl of 95% ethanol and mixing. The quantity of biofilms was measured by the absorbance of the samples at 570 nm. Untreated biofilms of *A. baumannii* and *C. albicans* were taken as controls corresponding to 100% of biofilms. All experiments were performed in duplicate and mean values and standard deviations (SD) were calculated.

### Membrane Permeability Assay

Microbial cell membrane damage provoked by the peptides was assayed with Live/Dead staining. *A. baumannii* and *C. albicans* were grown as for the antimicrobial activity assay and washed gently with PPB. Cells were resuspended in PPB, and diluted to OD_600_ = 0.1 and treated with peptides at various concentrations (as indicated in the Figures 3, 4 legends) at room temperature for 30 min. Untreated cells served as negative control. Cells were subsequently stained with 5 μM SYTO and 5 μM propidium iodide (PI). After 10 min incubation in the dark, 5 μl of the cell suspensions were spotted on microscope slide and covered with 2% (w/v) thin agar slices, and then observed with Olympus Fluoview FV 1000 confocal laser microscope at ×60 magnification using for SYTO9 488 nm laser for excitation, and 500–530 nm for emission. Excitation and emission wavelengths were 543 and 555–655 nm for PI, respectively. Sequential scanning was used to avoid crosstalk of the fluorescent dyes.

### Scanning Electron Microscopy

*A. baumannii* and *C. albicans* were grown and treated the same way as for the membrane permeability assay. Cells were then fixed with 2.5% (v/v) glutaraldehyde in phosphate buffered saline (PBS, pH 7.4). 5 μl of the above bacterial and yeast suspensions were spotted on a silicon disk coated with 0.01% Poly-L-Lysine. The filters were washed twice with PBS and gradually dehydrated with ethanol series (30, 50, 70, 80%, and three times 100% ethanol, each for minimum 30 min). The samples were dried with a critical point dryer (K850: Quorum Technologies Ltd.), followed by 12 nm gold coating and observed under a JEOL JSM-7100F/LV scanning electron microscope.

### *In Vitro* Transcription/Translation Assay

RTS 100 *E. coli* HY Kit (biotechrabbit Gmbh, Germany, catalog number: BR1400102) was used for the *in vitro* transcription/translation assay, according to the protocol. pIVEX2.3 control vector was used to produce GFP protein and to measure the translational efficiency. The amount of GFP produced was measured using a Hidex Plate Reader (Hidex Sense Microplate Reader with Plate Reader Software version 5064). The translational inhibitors, streptomycin and MtNCR247 as well as MtNCR001 without effect on translation (Farkas et al., 2014) were used as controls. All peptides and streptomycin were used at 100 µM concentration.

### Gel Retardation Assay

Interactions of peptides with DNA was visualized with gel retardation assay (Hsu et al., 2005). 100 ng Lambda DNA/HindIII marker (ThermoFisher Scientific) was incubated with 0, 5 or 10 µM synthetic NCRs or NCR derivatives in water for 30 min at room temperature and then loaded into 1% agarose gel. Electrophoresis of DNA fragments separated the mobile HindIII-digested DNA fragments while the aggregated DNA remained in the loading wells.

### Hemolysis Assay

Human blood was purchased from the Regional Blood Centre in Szeged. The use of human blood for the hemolysis assay has been authorized by the Regional Hungarian Ethics Committee and approved by the Ethics Review Sector of DG RTD (European Commission) in connection with EK’s ERC AdG SymBiotics. The cells from 10 ml of EDTA-blood were centrifuged at 1,500 × *g* for 1 min and washed several times in TBS buffer (10 mM Tris, pH = 7.2, 150 mM NaCl) until the supernatant became colorless. The cells were then resuspended in 12 ml TBS buffer and 100 μl of this cell suspension was incubated for 1 h at 37°C in the presence of NCR peptides (0.4–100 μM). The cells were centrifuged at 1,500 *g* for 1 min and the supernatants were transferred into sterile 96-well plates and the hemoglobin release was measured at OD_560_ (Hidex Sense Microplate Reader with Plate Reader Software version 5064). Cells in TBS buffer were used to determine baseline OD values, while 0.5% Triton X-100 (Serva) added at the same time as NCRs represented 100% of hemolysis. The hemolytic activity was calculated as % of red blood cell disruption relative to the positive control sample lysed with detergent Triton X-100.

### Serum Stability Assay

Peptides were incubated in 10% Fetal Bovine Serum at 37°C for 18 h. Full length NCRs were used at 25 μM, while short derivatives at 50 μM. The serum proteins and peptides were separated by sodium dodecyl sulfate-polyacrylamide gel electrophoresis (SDS-PAGE, 12% bis-tris gel, MES buffer pH 7.3) and visualized by Coomassie blue staining. NCRs with StrepII tag at their C-terminus were also detected by Western blot analysis using Precision Protein StrepTactin-HRP conjugate antibody (BioRad) and Clarity Western ECL (BioRad) for chemiluminescence detection according to the supplier.

## Results

### Antimicrobial Activity of NCR Peptides Requires Positive Net Charge and Depends on the Amino Acid Sequence

We investigated the antimicrobial activity of 78 NCR peptides from *M. truncatula* and 12 NCRs from other IRLC legumes as well as their shorter derivatives against *E. coli*, *S. enteritidis*, *P. aeruginosa*, *L. monocytogenes*, *S. aureus*, *E. faecalis*, *K. pneumoniae* and *A. baumannii* (Supplementary Table S1)*.* In the selection of the *M. truncatula* peptides, we considered their symbiotic expression levels but also their physicochemical properties. The antimicrobial activity of NCRs has so far been attributed to cationic peptides, with the exception of one anionic peptide. To investigate whether other anionic NCRs are antimicrobial, in addition to the cationic peptides, neutral and anionic NCRs were also included covering a broad range of peptides with different net charges (NC: from 7.8 to −10.2) and isoelectric points (pI: from 10.69 to 2.95). 50 peptides had positive net charge and pI > 7.0, while 28 peptides had negative net charge and pI < 7.0 (Supplementary Table S1A). Of the other legumes, only cationic NCR peptides were studied (Supplementary Table S1B).

The minimal bactericidal concentrations (MBCs) were determined after 3 h of treatment of ∼10^7^ log phase bacteria with 2-fold serial dilution of peptides from 25 µM to 0.8 µM. Only 25 of the 50 cationic *M. truncatula* peptides had activity at ≤25 µM (Supplementary Table S1). NCR073, NCR336 and NCR358 were the most active peptides capable of killing all tested bacteria. Of the 3 peptides, the less cationic peptides, NCR073 and NCR336 were somewhat more effective than the highly cationic NCR358. Interestingly, peptides with almost identical net charge and pI to the fully active NCR073 and NCR336 were inactive or only partially active, confirming high importance of the amino acid sequence. NCR377 and NCR645 could also kill all bacteria except *E. faecalis*. NCR280 and NCR700 were active against all strains except *S. aureus* and *E. faecalis.* NCR055 was also effective against six bacterial strains but not against *S. aureus* and *K. pneumonia*. Likewise, NCR384 was able to kill all tested bacteria except *E. faecalis* and *K. pneumoniae*. NCR183, NCR299, NCR471 and NCR520 were effective against five or four strains, while NCR135 against three strains. Three peptides were only active against two bacterial strains; NCR649 against *L*. *monocytogenes* and *A. baumannii*, NCR281 against *L. monocytogenes* and *P. aeruginosa* and NCR350 against *A. baumannii* and *P. aeruginosa*. NCR035 was only effective against *E. coli*, while NCR030 and NCR032 only against *A. baumannii*. None of the anionic NCR peptides affected the survival of the tested bacterial strains. However, the neutral peptide, NCR147 showed activity against four strains: *E. coli*, *P. aeruginosa*, *L. monocytogenes* and *A. baumannii*.

Of the other legumes, CaNCR13, CaNCR63, MsNCR443, MsNCR463, PsNCR349 and PsNCR352 had broad-spectrum activity, with the exception of MsNCR463, which was only effective against *P. aeruginosa* at 25 µM (Supplementary Table S1B). In MsNCR463 the positively charged amino acid residues accumulated at the C-terminal half of the molecule and we assumed that the relative inefficiency of the full length MsNCR463 might be caused by the N-terminal region. Therefore, we synthetized and tested two C-terminal derivatives of MsNCR463; MsNCR463_16-35_ and MsNCR463_17-30_. Likewise, nine lysine residues (K) were present in the C-terminal part of PsNCR349, that could be responsible for the antimicrobial activity, therefore, PsNCR349_26-50_ and PsNCR3493_31-50_ were also synthetized. On the contrary, the positively charged residues spread over the whole sequence in CaNCR63, indicating that likely the full-length sequence is necessary for the activity, though it could not be excluded that shorter derivatives of CaNCR63 were also active. From CaNCR63 we synthetized both the N-terminal sequence, CaNCR63_1-20_ and a C-terminal fragment, CaNCR63_15-34_. The MBC and the minimal fungicide concentration (MFC) of these peptides were determined against *A. baumannii* and *C. albicans* as examples of top bacterial and fungal pathogens on the WHO priority list (Table 1). All peptides were effective against both *A. baumannii* (MBCs: 1.6–6.25 µM) and *C. albicans* (MFCs: 1.6–25 µM), but the most active ones were the C-terminal derivatives of MsNCR463; MsNCR463_16-35_ and MsNCR463_17-30_ (MBC: 1.6 and 3.1 µM, MFC: 3.1 µM), which have the highest pI (11.79 and 12.44) of the tested NCRs. PsNCR349 and its C terminal derivative PsNCR349_26-50_ were similarly effective against *A. baumannii* (MBC: 3.125 µM) and *C. albicans* (MFC: 3.125 µM). The even shorter PsNCR349_31-50_, had slightly reduced activity against *A. baumannii* (MBC: 6.25 µM), but showed the best activity against *C. albicans* (MFC: 1.6 µM). The MBC value of the other peptides (CaNCR13, CaNCR63, CaNCR63_1-20_, CaNCR63_15-34_, MsNCR443, PsNCR349_31-50_, PsNCR352) was 6.25 µM.

In terms of pathogens, *E. faecalis* was the most resistant to NCRs, as only nine peptides (NCR055, NCR073, NCR183, NCR299, NCR336, NCR358, NCR520, GoNCR308, PsNCR351) were able to kill this Gram-positive bacterium. *K. pneumoniae* was sensitive to 13 NCRs, *S. aureus* to 18 NCRs, *S. enterica* to 21 NCRs, *L. monocytogenes* to 24 NCRs, *A. baumannii* to 27 NCRs, *E. coli* to 33 NCRs and *P. aeruginosa* to 36 NCRs (Supplementary Table S1).

### Physicochemical Properties Playing Role in the Antimicrobial Activity of NCRs

**FIGURE 1:**
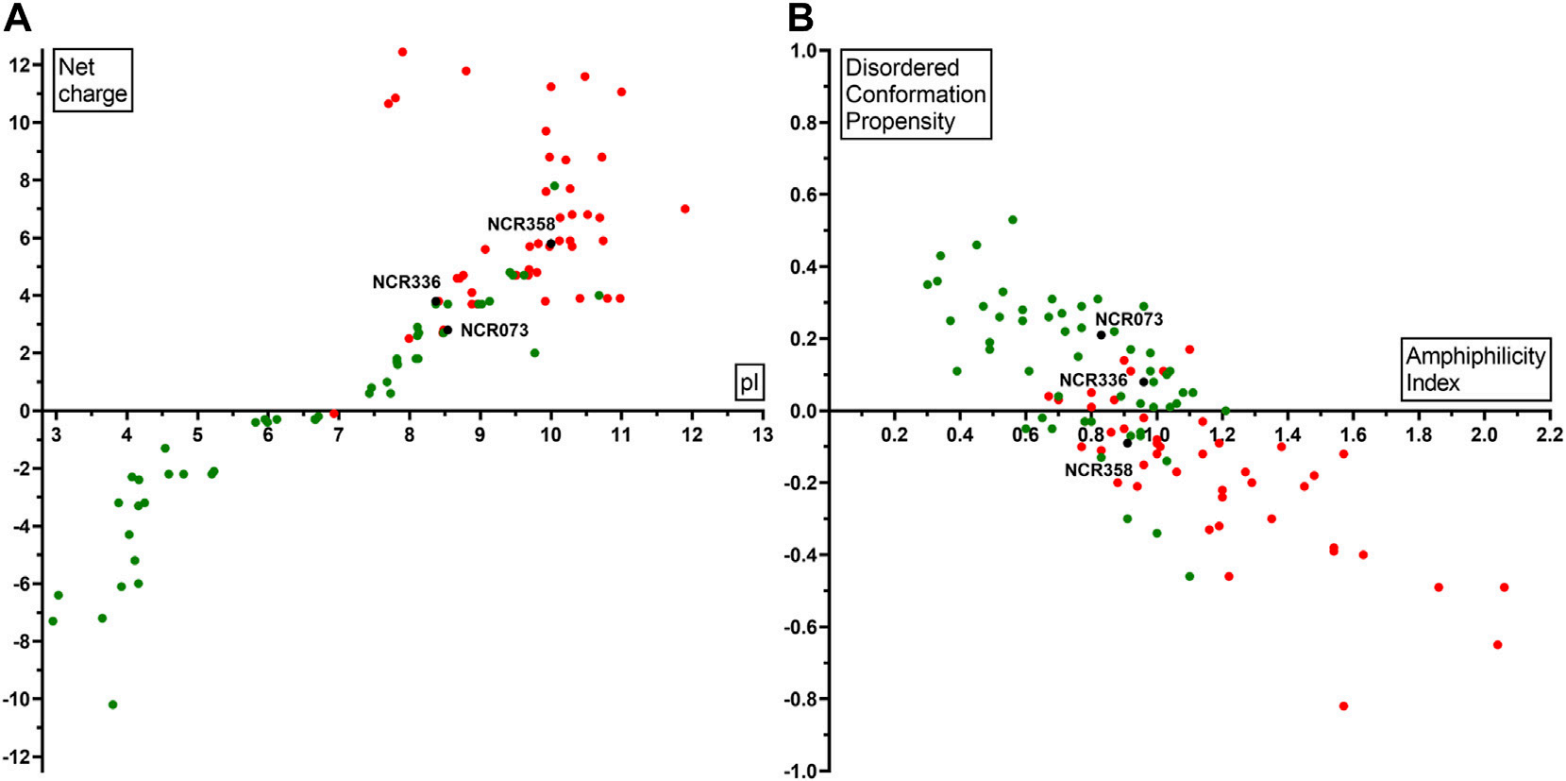
Physicochemical properties of NCRs in correlation with their antimicrobial activity **(A)** pI (*X* axis) and net charge (*Y* axis) **(B)** Amphiphilicity Index (*X* axis) and Disordered conformation propensity (*Y* axis) in correlation with the antimicrobial activity. Black dots show NCR073, NCR336, NCR358 which are active against all the eight tested pathogens. Red dots represent NCRs which are active against at least one pathogen. Green dots indicate the inactive NCRs.

Based on our experiments, the net charge and pI were not the only determinants of the antimicrobial activity. AMP predictions for all peptides by eight different tools (Supplementary Table S1) gave different and only partially overlapping results, and none were fully consistent with our experimental data, as several NCRs, with high pI and net charge predicted as AMPs, were inactive. Therefore, other physicochemical properties of the peptides may play a role in the antimicrobial property. We used DBAASP v3 database that provides tools for multifactor analysis of physicochemical properties of peptides (Pirtskhalava et al., 2021). The normalized hydrophobic moment, normalized hydrophobicity, net charge, pI, penetration depth, tilt angle, disordered conformation propensity, linear moment, propensity to *in vitro* aggregation, angle subtended by the hydrophobic residues, amphiphilicity index and propensity to PPII coil of all peptides is shown in Supplementary Table S1. Comparing these properties of the active and inactive peptides revealed significant differences in normalized hydrophobicity, net charge, pI, disordered conformation propensity, amphiphilicity index and to a lesser extent in linear moment and angle subtended by the hydrophobic residues (Supplementary Figure S1). The active NCRs are positively charged, cationic and disordered peptides, but less hydrophobic and more amphiphilic than the inactive peptides. The plot of pI and net charge and the amphiphilicity index and disordered conformation propensity of each peptide together with the activity shown in Figure 1. As it was evident from the antimicrobial tests, high pI and net charge were required for antimicrobial activity but in some cases they alone were insufficient (Figure 1A). NCRs with net charge higher than 2 can be antimicrobial, but NCRs with net charge higher than 5 were all active, with the exception of NCR192. Moreover, the disordered nature and higher amphiphilicity index of active NCRs (Figure 1B) indicate higher possibility for antimicrobial activity likely due to adoption of secondary structures at membrane-water interphases (Vishnepolsky and Pirtskhalava, 2014; Pirtskhalava et al., 2021).

### NCRs Degrade Biofilms of *C. albicans*

**FIGURE 2:**
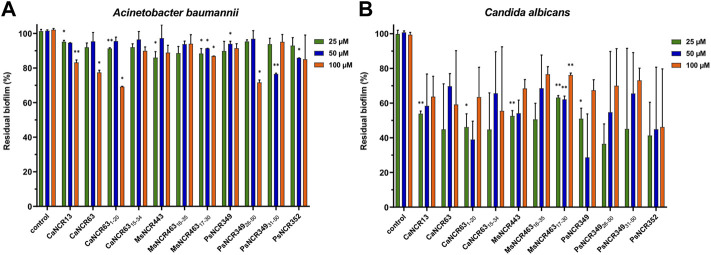
Effect of the peptides on biofilms of **(A)** *A. baumannii* and **(B)** *C. albicans*. The quantity of residual biofilms after overnight treatment with NCRs at 25, 50 or 100 μM compared to the untreated controls. The values are the means of two experiments, the bars indicate SD. *: *p* < 0.05, **: *p* < 0.01 as determined by one-way ANOVA.

Due to the protective effect of the matrix, pathogens in the biofilm are less accessible to antimicrobials and are therefore more resistant. As the biofilms are the main sources of persistent or recurrent infections, we tested whether NCRs could degrade the already formed biofilms. Biofilms of *A. baumannii* and *C. albicans* were treated overnight with 25, 50 and 100 µM concentrations of the peptides listed in Table 1. The quantity of biofilms remaining after the peptide treatments were compared to the control untreated biofilms (Figure 2). The peptides had only a mild effect on biofilms of *A. baumannii* while they were more effective in destroying the *C. albicans* biofilms reducing the biofilm quantity by 50–60%.

### NCR Peptides Cause Membrane Permeability, Morphological Changes and Cell Death

**FIGURE 3:**
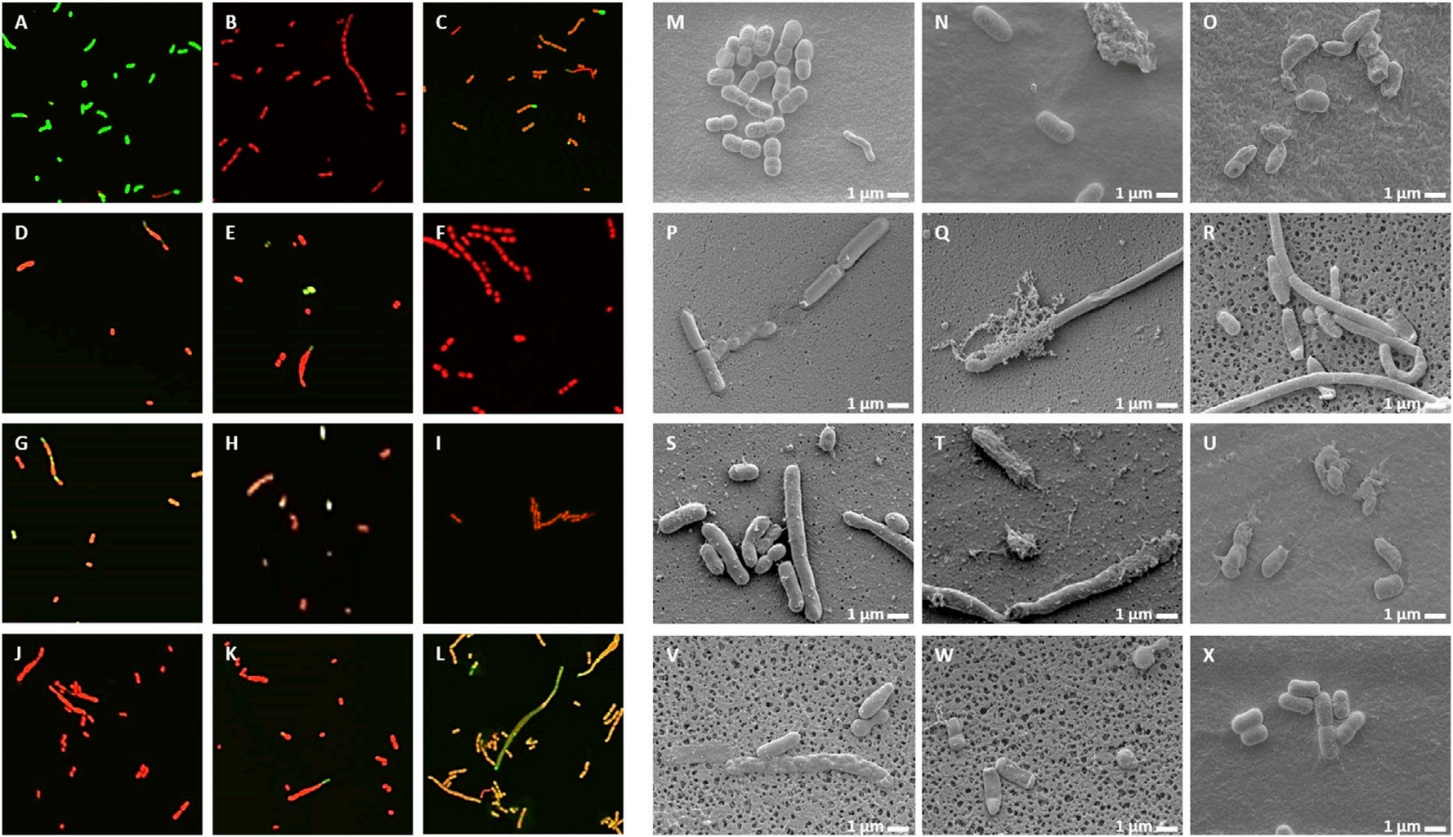
Viability, membrane permeability and morphology of *A. baumannii* cells after NCR treatment **(A–L)** SYTO9/PI staining observed with confocal microscope, **(M–X)** Cell morphology observed with SEM. **(A,M)** untreated control, **(B,N)** CaNCR13, 3.125 µM, **(C,O)** CaNCR63, 3.125 µM, **(D,P)** CaNCR63_1-20_, 3.125 µM, **(E,Q)** CaNCR63_15-34_, 3.125 µM, **(F,R)** MsNCR443, 3.125 µM, **(G,S)** MsNCR463_16-35_, 1.6 µM, **(H,T)** MsNCR463_17-30_, 3.125 µM, **(I,U)** PsNCR349, 1.6 µM, **(J,V)** PsNCR349_26-50_, 3.125 µM, **(K,W)** PsNCR349_31-50_, 3.125 µM, **(L,X)** PsNCR352, 1.6 µM.

**FIGURE 4:**
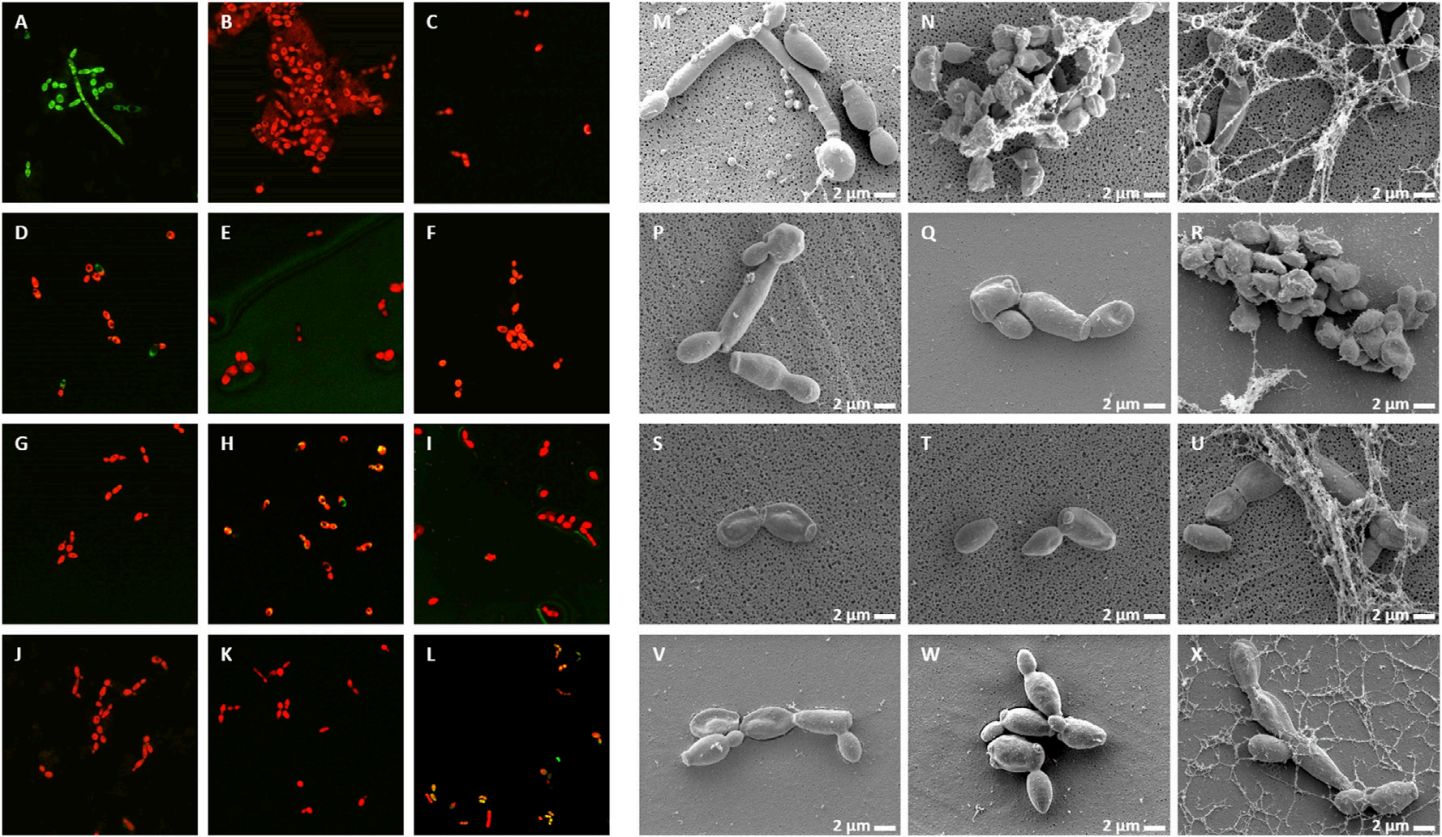
Viability, membrane permeability and morphology of *C. albicans* cells after NCR treatment **(A–L)** SYTO9/PI staining observed with confocal microscope, **(M–X)** Cell morphology observed with SEM **(A,M)** untreated control, **(B,N)** CaNCR13, 3.125 µM, **(C,O)** CaNCR63, 3.125 µM, **(D,P)** CaNCR63_1-20_, 25 μM, **(E,Q)** CaNCR63_15-34_, 3.125 µM, **(F,R)** MsNCR443, 3.125 µM, **(G,S)** MsNCR463_16-35_, 1.6 µM, **(H,T)** MsNCR463_17-30_, 1.6 µM, **(I,U)** PsNCR349, 1.6 µM, **(J,V)** PsNCR349_26-50_, 3.125 µM, **(K,W)** PsNCR349_31-50_, 3.125 µM, **(L,X)** PsNCR352, 1.6 µM.

Live/Dead staining of microbes with the membrane-permeable SYTO9 dye, which emits green fluorescence and propidium iodide (PI), which only enters the damaged dead cells and emits red fluorescence, allows investigation of membrane damage caused by the NCR peptides. In addition, morphological changes were also observed by scanning electron microscopy (SEM). *A. baumannii* (Figure 3) and *C. albicans* (Figure 4) cells were treated for 30 min with different concentrations of NCRs as indicated in the figure legends and then were either co-stained with SYTO9/PI dyes and observed under confocal microscopy, or the cells were fixed and observed with SEM. The untreated *A. baumannii* showed green fluorescence after the SYTO9/PI staining, while the treatment of *A. baumannii* with any of the peptides resulted in varying degrees of red fluorescence (Figure 3). Only red dead cells were observed after the treatment with CaNCR13, MsNCR443, PsNCR349_26-50_ and PsNCR349_31-50_ (Figures 3B,F,J,K). The few green, live cells observed among the red, dead cells might indicate a slower killing action of peptides CaNCR63, MsNCR463_16-35_, MsNCR463_17-30_ and PsNCR349 (Figures 3C,G–I), which can be even more pronounced in the case of PsNCR352 (Figure 3L) based on the generally yellowish staining of cells.

SEM revealed uniform size, morphology and smooth cell envelop of *A. baumannii* control cells (Figure 3M). This cell form was preserved in the case of PsNCR352 (Figure 3X) in line with the slower death indicated by the SYTO9/PI staining. The treatment by MsNCR463_16-35_ and MsNCR463_17-30_ caused budding of vesicles (Figures 3S,T). The treatment with CaNCR63, CaNCR63_1-20_ and CaNCR63_15-34_ caused rough cell surface, lysis and flattening of the cells (Figures 3O–Q). Only dead cells were visible after treatment with CaNCR13, PsNCR349 and PsNCR349_31-50_ (Figures 3N,U,W). Filament-like structures, observed also by confocal microscopy by attachment of cells at their poles, were formed in the presence of CaNCR63_15-34_, MsNCR443, MsNCR463_16-35_, MsNCR463_17-30_ and PsNCR349_26-50_ (Figures 3Q–V).

Treatment of *C. albicans* with any of these peptides resulted in PI staining of the cells while in the untreated control culture, all cells were alive and showed green fluorescence (Figure 4A–L). A few viable green cells were, however, observed in the CaNCR63_1-20_ sample (Figure 4D) in line with its highest MFC value. SEM observation revealed both yeast and hyphal forms in the control sample (Figure 4M). The CaNCR63_1-20_ treated sample was the most similar to the control (Figure 4P) where a few living cells were observed with confocal microscopy (Figure 4D). Flattening of cells was visible after CaNCR63, MsNCR463_16-35_, MsNCR463_17-30_, PsNCR349 and PsNCR352 treatments (Figures 4O,S,T,U,X) and aggregation of shrunken cells in a spider net like matrix in the samples treated with CaNCR13, CaNCR63, MsNCR443 and PsNCR352 (Figures 4N,O,R,X).

### NCR Peptides Have No or Only Weak Effects on Bacterial Translation

**FIGURE 5:**
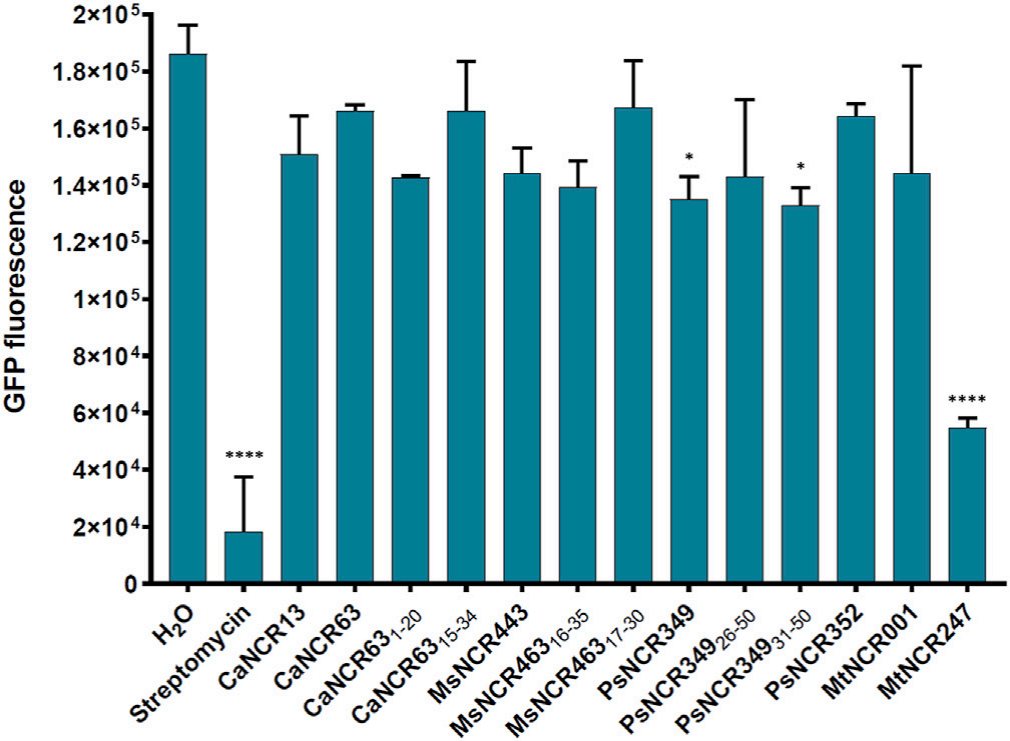
GFP production by *E. coli in vitro* coupled transcription-translation assay in the presence of 100 µM NCR or streptomycin. GFP fluorescence represents the average values of 2 technical parallels with their respective standard deviations (SD). *: *p* < 0.05, ****: *p* < 0.0001, as determined by one-way ANOVA.

There is growing evidence that AMPs in addition to their membrane interactions can also have intracellular bacterial targets and affect various cellular functions. Previously it was shown that MtNCR247 inhibits the bacterial translation, thus here we investigated if any of the peptides in Table 1 have similar functions. The effect of peptides was studied using an *E. coli in vitro* coupled transcription/translation assay, which measures the translational efficiency by GFP production (Figure 5). As controls, we included streptomycin and MtNCR247 as inhibitors of translation, as well as MtNCR001 without effect (Farkas et al., 2014). In agreement with previous data, streptomycin inhibited strongly and MtNCR247 to lesser extent the GFP production. The tested peptides resulted in GFP production comparable to the water and MtNCR001 controls indicating that these peptides have no significant effects on translation.

### NCR Peptides Can Form Complexes With DNA

**FIGURE 6:**
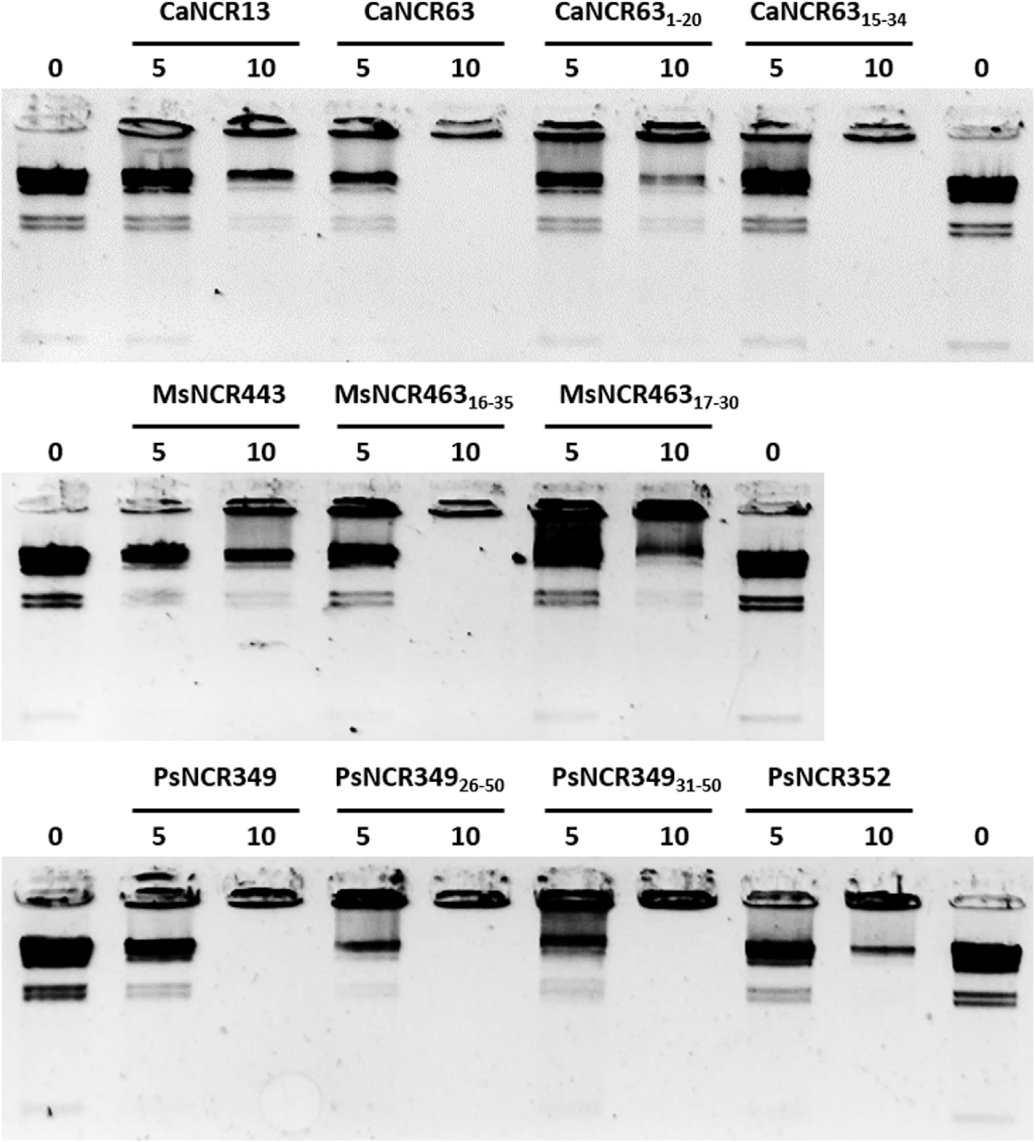
Aggregation of DNA by NCR peptides. Lambda HindIII digested DNA was incubated without (0) or with NCR peptides at 5 or 10 μM concentration.

In the natural symbiotic system NCRs provoke amplification of bacterial DNA via endoreduplication cycles (Mergaert et al., 2006). While the functions of NCRs in this process is still unknown we investigated whether the peptides could interact with DNA using HindIII-digested λ DNA as test molecule (Figure 6). The peptides were added to the DNA at 5 μM and 10 μM concentrations for 30 min and then loaded into agarose gel. Separation of the λ HindIII-fragments by gel electrophoresis occurred in the control untreated samples, while the DNA remained in the wells in the presence of 10 μM CaNCR63, CaNCR63_15-34_, MsNCR463_16-35_, PsNCR349, PsNCR349_26-50_ and PsNCR349_31-50_. While these peptides aggregated the DNA fragments, CaNCR13, MsNCR443 and PsNCR352 did not. The N-terminal fragment of CaNCR63, CaNCR63_1-20_ was less efficient than either the full-length peptide or the C-terminal CaNCR63_15-34_, which retained completely the DNA-binding ability. CaNCR63_1-20_ and CaNCR63_15-34_ have similar pI and net charge values, indicating the contribution of the sequence downstream of the 15th amino acid to the DNA-binding. In PsNCR349 the positively charged amino acid residues are at the C-terminal part, accordingly the sequence from 31 to 50 was sufficient for aggregation of DNA. PsNCR352 was less active in DNA-binding than PsNCR349, which may be due to its lower net charge (6.80). MsNCR463_17-30_ was less efficient than MsNCR463_16-35_ indicating that the last five amino acids are necessary to the DNA-interaction.

### Antimicrobial NCRs Do Not Provoke Notable Hemolysis and Are Stable in the Presence of Serum

While AMPs can be very effective against microbes, they should not be toxic for human cells. The potential hemolytic activity of the legume peptides listed in Table 1 was tested in hemolysis assay (Jenei et al., 2020). Human red blood cells were co-incubated with the peptides used in two-fold dilutions from 100 to 0.2 µM or with 0.5% Triton X-100 provoking complete hemolysis. Disruption of red blood cells was measured by spectrophotometry and compared to samples lysed with the detergent Triton X-100 (Supplementary Figure S2). None of the peptides caused hemolysis in the studied concentration range, except MsNCR465, provoking mild hemolysis at 50 and 100 μM concentrations, far above the MBCs of this peptide.

We also tested if NCRs remain stable in the presence of serum. 18 antimicrobial NCRs and NCR derivatives were incubated overnight at 37°C with Fetal Bovine Serum (FBS). The amount of peptides with or without FBS was comparable based on Coomassie Blue staining of SDS-PAGE gels (Supplementary Figure S3). To confirm the results, NCR183 and NCR055 carrying a StrepII tag at their C-terminus were also visualized by Western blot analysis using anti-StrepII-tag antibody and in line with the results of Coomassie Blue staining both of them were stable in the presence of FBS.

## Discussion

Species belonging to the IRLC clade of legumes likely produce hundreds of thousands of NCR peptides in symbiosis mostly having roles in terminal differentiation of their rhizobium bacterium partner. In recent years, many studies have referred to NCR peptides as AMPs being effective against several pathogens (Mergaert, 2018; Jenei et al., 2020; Lima et al., 2020; Velivelli et al., 2020; Isozumi et al., 2021). Until now, only *M. truncatula* NCRs were tested and except for the anionic NCR211 (Kim et al., 2015) all NCRs with antimicrobial properties were cationic. MtNCRs above pI 9.5 were also effective against *Candida* species (Ördögh et al., 2014; Szerencsés et al., 2021). According to the sequence analysis, NCRs represent a novel class of AMPs (Lima et al., 2020).

In this work we addressed three main questions: i) are all cationic NCRs antimicrobial agents? ii) do the non-cationic NCRs have antimicrobial activities? and iii) do cationic NCRs from other IRLC legumes have antimicrobial activities?

The first and second questions were answered by studying 78 MtNCR peptides varying in their charge from −10.2 to 7.8 and isoelectric points from 2.95 to 10.69 against eight pathogenic bacteria including ESKAPE strains. 50 peptides had pI above 7.0, and 25 of them had antibacterial activities. Among the eight AMP prediction tools used in this study, CS-AMPPred developed to predict the antimicrobial activity of cysteine-stabilized peptides (Porto et al., 2012) and CAMP_R3_ using a large dataset of AMPs (Waghu et al., 2016; Waghu and Idicula-Thomas, 2020), were relatively the most accurate in finding active NCRs (Supplementary Table S1). However, the measured efficiency of these AMP predictions appears to be lower for NCRs than for other AMPs. The most important determinant of NCR activity was the high net charge and all tested NCRs with net charge higher than 5 (except NCR192) were active against at least one pathogenic bacteria. Previous work demonstrated the importance of pI values above 9.5 in antifungal activities (Ördögh et al., 2014) and here we show that pI > 9.5 is not an absolute requirement for the antibacterial effect as 12 peptides were effective with lower pI values (7.99–9.49), as was the neutral NCR147 peptide (pI 6.93). However, high positive charge and pI alone did not guarantee activity, as several peptides with such properties were inactive. The amino acid sequence and thereby other physicochemical properties of peptides could also contribute and be crucial for the antimicrobial activity. For example, the disordered conformation and higher amphiphilicity index show correlation with the NCRs’ antimicrobial activity. It will need further research to find out the structure-function relationship, why certain peptides with similar physicochemical properties were active or inactive in our screen. Three peptides were effective against all bacterial strains, eleven NCRs had broad range of activities, while seven NCRs could only kill one or two strains and three of them only *A. baumannii*. Having peptides with either broad or highly specific activities could be important for therapeutic applications.

One drawback of using cationic AMPs is their sensitivity to divalent cations; Ca^2+^, Mg^2+^, which can decrease their activity by inhibiting their interactions with microbial membranes. Therefore, non-cationic NCRs, likely with different modes of action, could be even more suited for therapeutic applications. We have tested 28 non-cationic NCR peptides and except for the neutral NCR147 (pI: 6.93, NC: −0.1) none of the peptides had antimicrobial activity. In this test, NCR211, with reported antibacterial activity against the symbiotic bacterium partner (Kim et al., 2015), was also inactive and even by repeating the described conditions the antibacterial activity of NCR211 could not be confirmed. It will be interesting to find more neutral NCRs with antimicrobial activities and to discover the mode of their killing actions.

To answer the third question, by analyzing 12 cationic NCRs from four legume species, we confirmed that NCRs from different IRLC legumes have antimicrobial activities. The activity of five broad spectrum IRLC NCRs and their six derivatives was tested against *A. baumannii* and *C. albicans.* All peptides were effective against *A. baumannii* and all except one against *C. albicans* with 1.6–6.25 µM MBC/MFC (Table 1). Moreover, the peptides were able to degrade *C. albicans* biofilm that can have a particular importance in therapeutic applications (Figure 1). Although understanding their exact mode of action has not been the subject of the present work and requires further research, this work indicates that their antimicrobial effects are primarily based on their interaction with microbial membranes. Within 30 min both *A. baumannii* and *C. albicans* cells were killed as it was demonstrated by confocal microscopy and SEM (Figures 2, 3). However, it is likely that the killing action of certain NCRs can even be faster as it was observed for other MtNCRs (Jenei et al., 2020). The NCRs may also enter the cells and have intracellular targets. In our experiments the tested NCRs had no significant effect on the *in vitro* coupled transcription/translation (Figure 4), but they provoked aggregation of DNA with different efficacy (Figure 5). These NCRs even at high 100 μM did not provoke hemolysis of human blood cells (Figure 6).

Our work confirmed that cationic NCRs from other IRLC legumes also have antimicrobial activities, of which MsNCR463_17-30_ and PsNCR3493_31-50_ are outstanding with their efficiency and short length (14 and 20 amino acids), which makes their synthesis cost effective. We proved with MtNCR147 that neutral NCRs can be antimicrobial agents as well. Thus, the 4000 IRLC species can provide an uncountable source of antimicrobial peptides. The peptides selected for this study provide already a list of potential candidates for further drug development and clinical trials.

## Data Availability

The original contributions presented in the study are included in the article/Supplementary Material, further inquiries can be directed to the corresponding author.

## Supplementary Material

Supplementary Figure S1 Physicochemical properties of active and inactive NCRs calculated by DBAASP v3 (normalized hydrophobic moment, normalized hydrophobicity, penetration depth, tilt angle, disordered conformation propensity, linear moment, propensity to *in vitro* aggregation, angle subtended by the hydrophobic residues, amphiphilicity index, propensity to PPII coil) and PepCalc.com (net charge, pI). Active NCRs have antimicrobial activity against at least one pathogen. Graphs represent mean ± SD. *: *p* < 0.05, ****: *p* < 0.0001, as determined by *t*-test.

Supplementary Figure S2 Hemolytic activity of NCRs. Human red blood cells were treated with different NCRs (0.4–100 µM). Hemolysis of red blood cells with 0.5% Triton-X represents 100% hemolytic activity.

## References

[B1] Balogh E. Mosolygó T. Tiricz H. Szabó Á. Karai A. Kerekes F. (2014). Anti-Chlamydial Effect of Plant Peptides. Acta Microbiol. Immunol. Hung. 61, 229–239. 10.1556/AMicr.61.2014.2.12 24939689

[B2] Bhatia P. Sharma A. George A. J. Anvitha D. Kumar P. Dwivedi V. P. (2021). Antibacterial Activity of Medicinal Plants Against ESKAPE: An Update. Heliyon 7, e06310. 10.1016/j.heliyon.2021.e06310 33718642 PMC7920328

[B3] Büyükkiraz M. E. Kesmen Z. (2021). Antimicrobial Peptides (AMPs): A Promising Class of Antimicrobial Compounds. J. Appl. Microbiol. 132, 1573–1596. 10.1111/jam.15314 34606679

[B4] Campos M. L. de Souza C. M. de Oliveira K. B. S. Dias S. C. Franco O. L. (2018). The Role of Antimicrobial Peptides in Plant Immunity. J. Exp. Bot. 69, 4997–5011. 10.1093/jxb/ery294 30099553

[B5] Cesare G. B. D. Cristy S. A. Garsin D. A. Lorenz M. C. (2020). Antimicrobial Peptides: A New Frontier in Antifungal Therapy. mBio 11 (6), e02123–20. 10.1128/mBio.02123-20 33144376 PMC7642678

[B6] Duan L. Li S.-J. Su C. Sirichamorn Y. Han L.-N. Ye W. (2021). Phylogenomic Framework of the IRLC Legumes (*Leguminosae* Subfamily *Papilionoideae*) and Intercontinental Biogeography of Tribe *Wisterieae* . Mol. Phylogenet. Evol. 163, 107235. 10.1016/j.ympev.2021.107235 34146677

[B7] Egorov T. A. Odintsova T. I. Pukhalsky V. A. Grishin E. V. (2005). Diversity of Wheat Anti-Microbial Peptides. Peptides 26, 2064–2073. 10.1016/j.peptides.2005.03.007 16269343

[B8] Farkas A. Maróti G. Dürgő H. Györgypál Z. Lima R. M. Medzihradszky K. F. (2014). *Medicago Truncatula* Symbiotic Peptide NCR247 Contributes to Bacteroid Differentiation through Multiple Mechanisms. Proc. Natl. Acad. Sci. 111, 5183–5188. 10.1073/pnas.1404169111 24706863 PMC3986156

[B9] Farkas A. Maróti G. Kereszt A. Kondorosi É. (2017). Comparative Analysis of the Bacterial Membrane Disruption Effect of Two Natural Plant Antimicrobial Peptides. Front. Microbiol. 8, 51. 10.3389/fmicb.2017.00051 28167938 PMC5253368

[B10] Fernando S. A. Gray T. J. Gottlieb T. (2017). Healthcare-Acquired Infections: Prevention Strategies. Int. Med. J. 47, 1341–1351. 10.1111/imj.13642 29224205

[B11] Hancock R. E. (1997). Peptide Antibiotics. Lancet 349, 418–422. 10.1016/S0140-6736(97)80051-7 9033483

[B12] Harding C. M. Hennon S. W. Feldman M. F. (2018). Uncovering the Mechanisms of *Acinetobacter Baumannii* Virulence. Nat. Rev. Microbiol. 16, 91–102. 10.1038/nrmicro.2017.148 29249812 PMC6571207

[B13] Hsu C.-H. Chen C. Jou M.-L. Lee A. Y.-L. Lin Y.-C. Yu Y.-P. (2005). Structural and DNA-Binding Studies on the Bovine Antimicrobial Peptide, Indolicidin: Evidence for Multiple Conformations Involved in Binding to Membranes and DNA. Nucleic. Acids. Res. 33, 4053–4064. 10.1093/nar/gki725 16034027 PMC1179735

[B14] Isozumi N. Masubuchi Y. Imamura T. Mori M. Koga H. Ohki S. (2021). Structure and Antimicrobial Activity of NCR169, a Nodule-Specific Cysteine-Rich Peptide of *Medicago Truncatula* . Sci. Rep. 11, 9923. 10.1038/s41598-021-89485-w 33972675 PMC8110993

[B15] Ištvánek J. Jaroš M. Křenek A. Řepková J. (2014). Genome Assembly and Annotation for Red Clover (*Trifolium Pratense; Fabaceae*). Am. J. Bot. 101, 327–337. 10.3732/ajb.1300340 24500806

[B16] Jenei S. Tiricz H. Szolomájer J. Tímár E. Klement É. Al Bouni M. A. (2020). Potent Chimeric Antimicrobial Derivatives of the *Medicago Truncatula* NCR247 Symbiotic Peptide. Front. Microbiol. 11, 270. 10.3389/fmicb.2020.00270 32153547 PMC7047876

[B17] Kanugala S. Jinka S. Puvvada N. Banerjee R. Kumar C. G. (2019). Phenazine-1-Carboxamide Functionalized Mesoporous Silica Nanoparticles as Antimicrobial Coatings on Silicone Urethral Catheters. Sci. Rep. 9, 6198. 10.1038/s41598-019-42722-9 30996286 PMC6470230

[B18] Kim M. Chen Y. Xi J. Waters C. Chen R. Wang D. (2015). An Antimicrobial Peptide Essential for Bacterial Survival in the Nitrogen-Fixing Symbiosis. Proc. Natl. Acad. Sci. 112, 15238–15243. 10.1073/pnas.1500123112 26598690 PMC4679048

[B19] Kyriakidis I. Vasileiou E. Pana Z. D. Tragiannidis A. (2021). *Acinetobacter Baumannii* Antibiotic Resistance Mechanisms. Pathogens 10, 373. 10.3390/pathogens10030373 33808905 PMC8003822

[B20] Li J. Hu S. Jian W. Xie C. Yang X. (2021). Plant Antimicrobial Peptides: Structures, Functions, and Applications. Bot. Stud. 62, 5. 10.1186/s40529-021-00312-x 33914180 PMC8085091

[B21] Lima R. M. Kylarová S. Mergaert P. Kondorosi É. (2020). Unexplored Arsenals of Legume Peptides with Potential for Their Applications in Medicine and Agriculture. Front. Microbiol. 11, 1307. 10.3389/fmicb.2020.01307 32625188 PMC7314904

[B22] Magana M. Pushpanathan M. Santos A. L. Leanse L. Fernandez M. Ioannidis A. (2020). The Value of Antimicrobial Peptides in the Age of Resistance. Lancet Infect. Dis. 20, e216–e230. 10.1016/S1473-3099(20)30327-3 32653070

[B23] Maróti G. Downie J. A. Kondorosi É. (2015). Plant Cysteine-Rich Peptides that Inhibit Pathogen Growth and Control Rhizobial Differentiation in Legume Nodules. Curr. Opin. Plant Biol. 26, 57–63. 10.1016/j.pbi.2015.05.031 26116977

[B24] Mergaert P. Nikovics K. Kelemen Z. Maunoury N. Vaubert D. Kondorosi A. (2003). A Novel Family in *Medicago Truncatula* Consisting of More Than 300 Nodule-Specific Genes Coding for Small, Secreted Polypeptides with Conserved Cysteine Motifs. Plant Physiol. 132, 161–173. 10.1104/pp.102.018192 12746522 PMC166962

[B25] Mergaert P. (2018). Role of Antimicrobial Peptides in Controlling Symbiotic Bacterial Populations. Nat. Prod. Rep. 35, 336–356. 10.1039/C7NP00056A 29393944

[B26] Mergaert P. Uchiumi T. Alunni B. Evanno G. Cheron A. Catrice O. (2006). Eukaryotic Control on Bacterial Cell Cycle and Differentiation in the *Rhizobium*–Legume Symbiosis. Proc. Natl. Acad. Sci. 103, 5230–5235. 10.1073/pnas.0600912103 16547129 PMC1458823

[B27] Mikuláss K. R. Nagy K. Bogos B. Szegletes Z. Kovács E. Farkas A. (2016). Antimicrobial Nodule-Specific Cysteine-Rich Peptides Disturb the Integrity of Bacterial Outer and Inner Membranes and Cause Loss of Membrane Potential. Ann. Clin. Microbiol. Antimicrob. 15, 43. 10.1186/s12941-016-0159-8 27465344 PMC4964015

[B28] Montiel J. Downie J. A. Farkas A. Bihari P. Herczeg R. Bálint B. (2017). Morphotype of Bacteroids in Different Legumes Correlates with the Number and Type of Symbiotic NCR Peptides. Proc. Natl. Acad. Sci. 114, 5041–5046. 10.1073/pnas.1704217114 28438996 PMC5441718

[B29] Moser D. Biere K. Han B. Hoerl M. Schelling G. Choukér A. (2021). COVID-19 Impairs Immune Response to *Candida Albicans* . Front. Immunol. 12, 640644. 10.3389/fimmu.2021.640644 33717195 PMC7953065

[B30] Nallu S. Silverstein K. A. T. Samac D. A. Bucciarelli B. Vance C. P. VandenBosch K. A. (2013). Regulatory Patterns of a Large Family of Defensin-Like Genes Expressed in Nodules of *Medicago Truncatula* . PLoS One 8, e60355. 10.1371/journal.pone.0060355 23573247 PMC3613412

[B31] Oliveira L. V. N. Wang R. Specht C. A. Levitz S. M. (2021). Vaccines for Human Fungal Diseases: Close but Still a Long Way to Go. npj Vaccines 6, 33. 10.1038/s41541-021-00294-8 33658522 PMC7930017

[B32] Ördögh L. Vörös A. Nagy I. Kondorosi É. Kereszt A. (2014). Symbiotic Plant Peptides Eliminate *Candida Albicans* Both *In Vitro* and in an Epithelial Infection Model and Inhibit the Proliferation of Immortalized Human Cells. Biomed. Res. Int. 2014, 1–9. 10.1155/2014/320796 PMC4163382 25243129

[B33] Pirtskhalava M. Amstrong A. A. Grigolava M. Chubinidze M. Alimbarashvili E. Vishnepolsky B. (2021). DBAASP v3: Database of Antimicrobial/Cytotoxic Activity and Structure of Peptides as a Resource for Development of New Therapeutics. Nucleic Acids Res. 49, D288–D297. 10.1093/nar/gkaa991 33151284 PMC7778994

[B34] Porto W. F. Ferreira K. C. V. Ribeiro S. M. Franco O. L. (2022). Sense the Moment: A Highly Sensitive Antimicrobial Activity Predictor Based on Hydrophobic Moment. Biochim. Biophys. Acta Gen. Subj. 1866, 130070. 10.1016/j.bbagen.2021.130070 34953809

[B35] Porto W. F. Pires Á. S. Franco O. L. (2012). CS-AMPPred: An Updated SVM Model for Antimicrobial Activity Prediction in Cysteine-Stabilized Peptides. PLoS One 7, e51444. 10.1371/journal.pone.0051444 23240023 PMC3519874

[B36] Rangel K. Chagas T. P. G. De-Simone S. G. (2021). *Acinetobacter Baumannii* Infections in Times of COVID-19 Pandemic. Pathogens 10, 1006. 10.3390/pathogens10081006 34451470 PMC8399974

[B37] Ribeiro C. W. Baldacci-Cresp F. Pierre O. Larousse M. Benyamina S. Lambert A. (2017). Regulation of Differentiation of Nitrogen-Fixing Bacteria by Microsymbiont Targeting of Plant Thioredoxin S1. Curr. Biol. 27, 250–256. 10.1016/j.cub.2016.11.013 28017611

[B38] Roy P. Achom M. Wilkinson H. Lagunas B. Gifford M. L. (2020). Symbiotic Outcome Modified by the Diversification from 7 to over 700 Nodule-Specific Cysteine-Rich Peptides. Genes 11, 348. 10.3390/genes11040348 PMC7230169 32218172

[B39] Shabab M. Arnold M. F. F. Penterman J. Wommack A. J. Bocker H. T. Price P. A. (2016). Disulfide Cross-Linking Influences Symbiotic Activities of Nodule Peptide NCR247. Proc. Natl. Acad. Sci. 113, 10157–10162. 10.1073/pnas.1610724113 27551097 PMC5018749

[B40] Szerencsés B. Gácser A. Endre G. Domonkos I. Tiricz H. Vágvölgyi C. (2021). Symbiotic NCR Peptide Fragments Affect the Viability, Morphology and Biofilm Formation of *Candida* Species. Int. J. Mol. Sci. 22, 3666. 10.3390/ijms22073666 33915930 PMC8037406

[B41] Tam J. Wang S. Wong K. Tan W. (2015). Antimicrobial Peptides from Plants. Pharmaceuticals 8, 711–757. 10.3390/ph8040711 26580629 PMC4695807

[B42] Tiricz H. Szűcs A. Farkas A. Pap B. Lima R. M. Maróti G. (2013). Antimicrobial Nodule-Specific Cysteine-Rich Peptides Induce Membrane Depolarization-Associated Changes in the Transcriptome of *Sinorhizobium Meliloti* . Appl. Environ. Microbiol. 79, 6737–6746. 10.1128/AEM.01791-13 23995935 PMC3811505

[B43] Van de Velde W. Zehirov G. Szatmari A. Debreczeny M. Ishihara H. Kevei Z. (2010). Plant Peptides Govern Terminal Differentiation of Bacteria in Symbiosis. Science 327, 1122–1126. 10.1126/science.1184057 20185722

[B44] Velivelli S. L. S. Czymmek K. J. Li H. Shaw J. B. Buchko G. W. Shah D. M. (2020). Antifungal Symbiotic Peptide NCR044 Exhibits Unique Structure and Multifaceted Mechanisms of Action that Confer Plant Protection. Proc. Natl. Acad. Sci. 117, 16043–16054. 10.1073/pnas.2003526117 32571919 PMC7354933

[B45] Veltri D. Kamath U. Shehu A. (2018). Deep Learning Improves Antimicrobial Peptide Recognition. Bioinformatics 34, 2740–2747. 10.1093/bioinformatics/bty179 29590297 PMC6084614

[B46] Vishnepolsky B. Pirtskhalava M. (2014). Prediction of Linear Cationic Antimicrobial Peptides Based on Characteristics Responsible for Their Interaction with the Membranes. J. Chem. Inf. Model. 54, 1512–1523. 10.1021/ci4007003 24730612 PMC4038373

[B47] Waghu F. H. Barai R. S. Gurung P. Idicula-Thomas S. (2016). CAMPR3: A Database on Sequences, Structures and Signatures of Antimicrobial Peptides. Nucleic Acids Res. 44, D1094–D1097. 10.1093/nar/gkv1051 26467475 PMC4702787

[B48] Waghu F. H. Idicula‐Thomas S. (2020). Collection of Antimicrobial Peptides Database and its Derivatives: Applications and Beyond. Protein Sci. 29, 36–42. 10.1002/pro.3714 31441165 PMC6933839

[B49] Wang G. Li X. Wang Z. (2016). APD3: The Antimicrobial Peptide Database as a Tool for Research and Education. Nucleic Acids Res. 44, D1087–D1093. 10.1093/nar/gkv1278 26602694 PMC4702905

[B50] Yamaguchi Y. Huffaker A. (2011). Endogenous Peptide Elicitors in Higher Plants. Curr. Opin. Plant Biol. Biot. Interact. 14, 351–357. 10.1016/j.pbi.2011.05.001 21636314

[B51] Zasloff M. (2002). Antimicrobial Peptides of Multicellular Organisms. Nature 415, 389–395. 10.1038/415389a 11807545

