# Supplementary figures and images for: Legume Plant Peptides as Sources of Novel Antimicrobial Molecules Against Human Pathogens
Source: Front Mol Biosci. 2022 Jun 9;9:870460. doi: 10.3389/fmolb.2022.870460 (PMC9218685; doi:10.3389/fmolb.2022.870460)

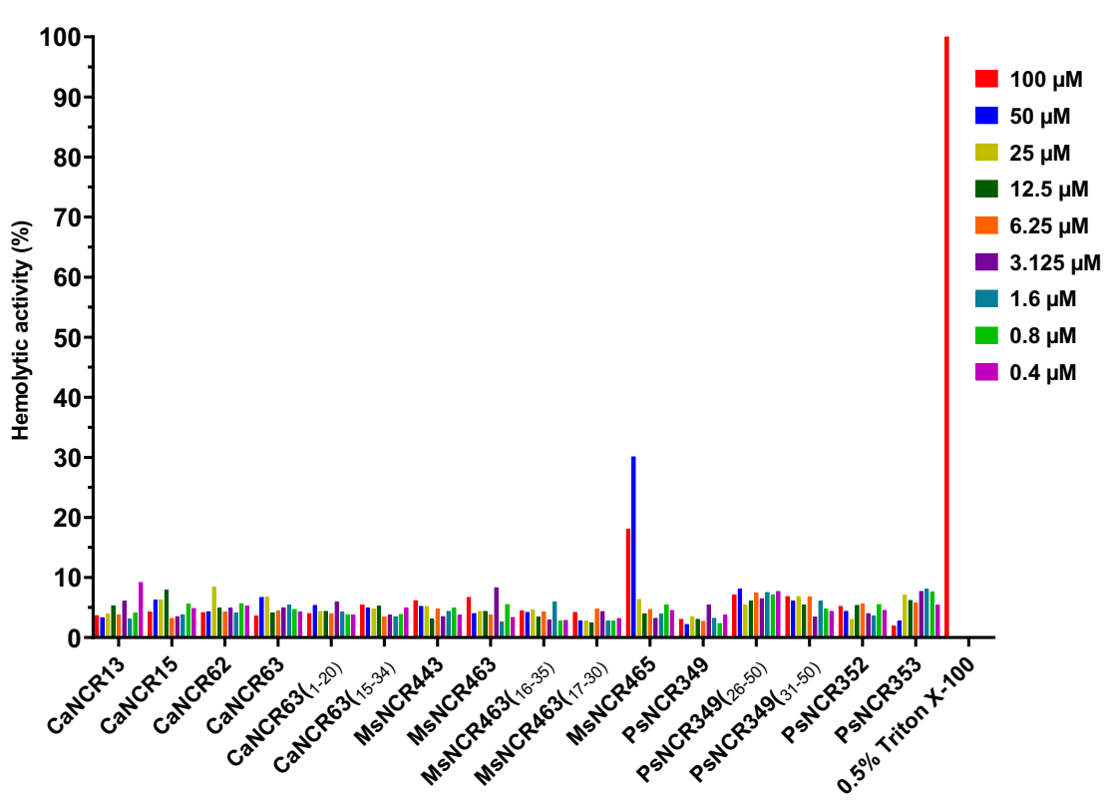

Supplement: Supplementary file 1 [file Image2.TIF]

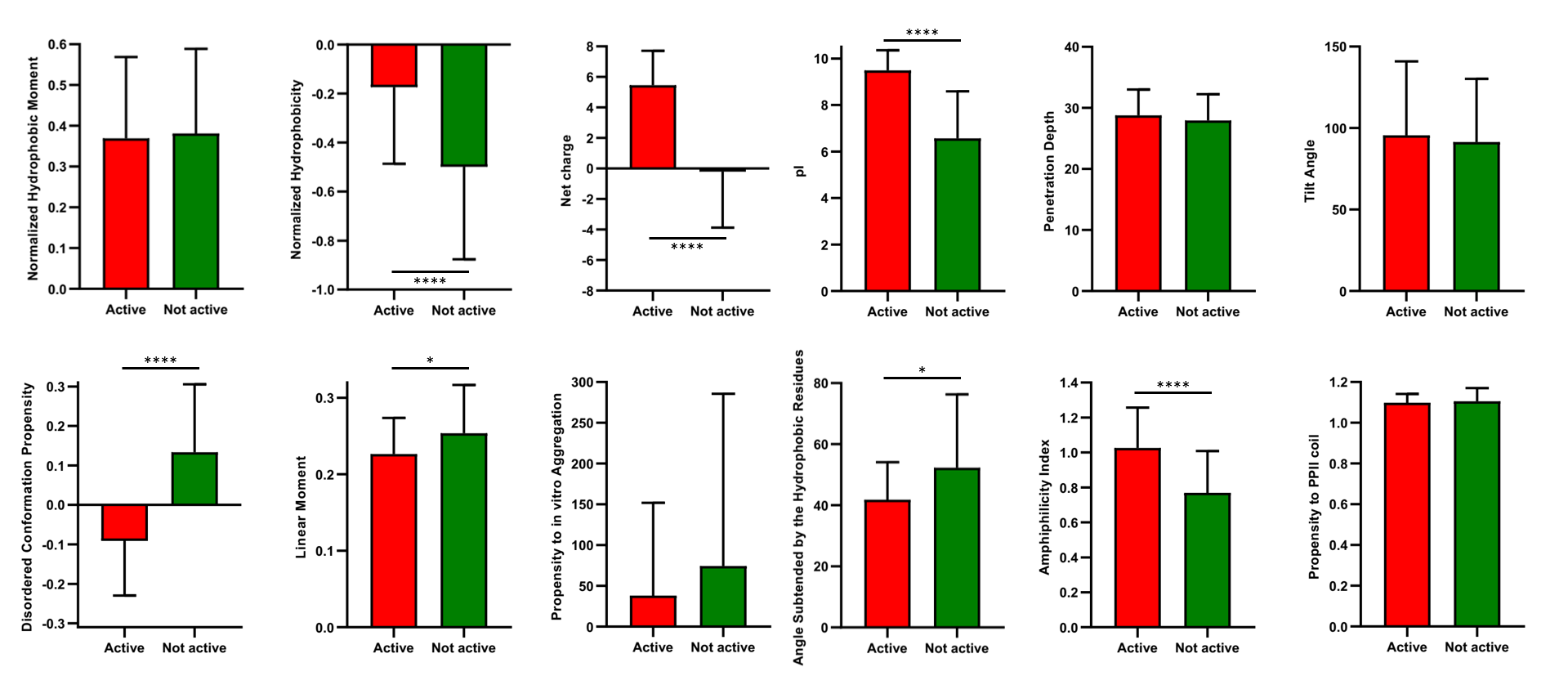

Supplement: Supplementary file 2 [file Image1.TIF]
