# Supplementary material for: Legume Plant Peptides as Sources of Novel Antimicrobial Molecules Against Human Pathogens
Source: Front Mol Biosci. 2022 Jun 9;9:870460. doi: 10.3389/fmolb.2022.870460 (PMC9218685; doi:10.3389/fmolb.2022.870460)

SUPPLEMENTARY FIGURE S3 Serum stability assay. Full length NCRs, StrepII-tagged NCR peptides (25 µM) and shorter NCR derivatives (50 µM) were incubated in water or in 10% Fetal Bovine Serum (FBS) for 18 hours at 37°C. Serum proteins and the peptides were separated by SDS-Page and visualized by Coomassie blue staining or in the case of StrepII-tagged NCRs also with Western blot.

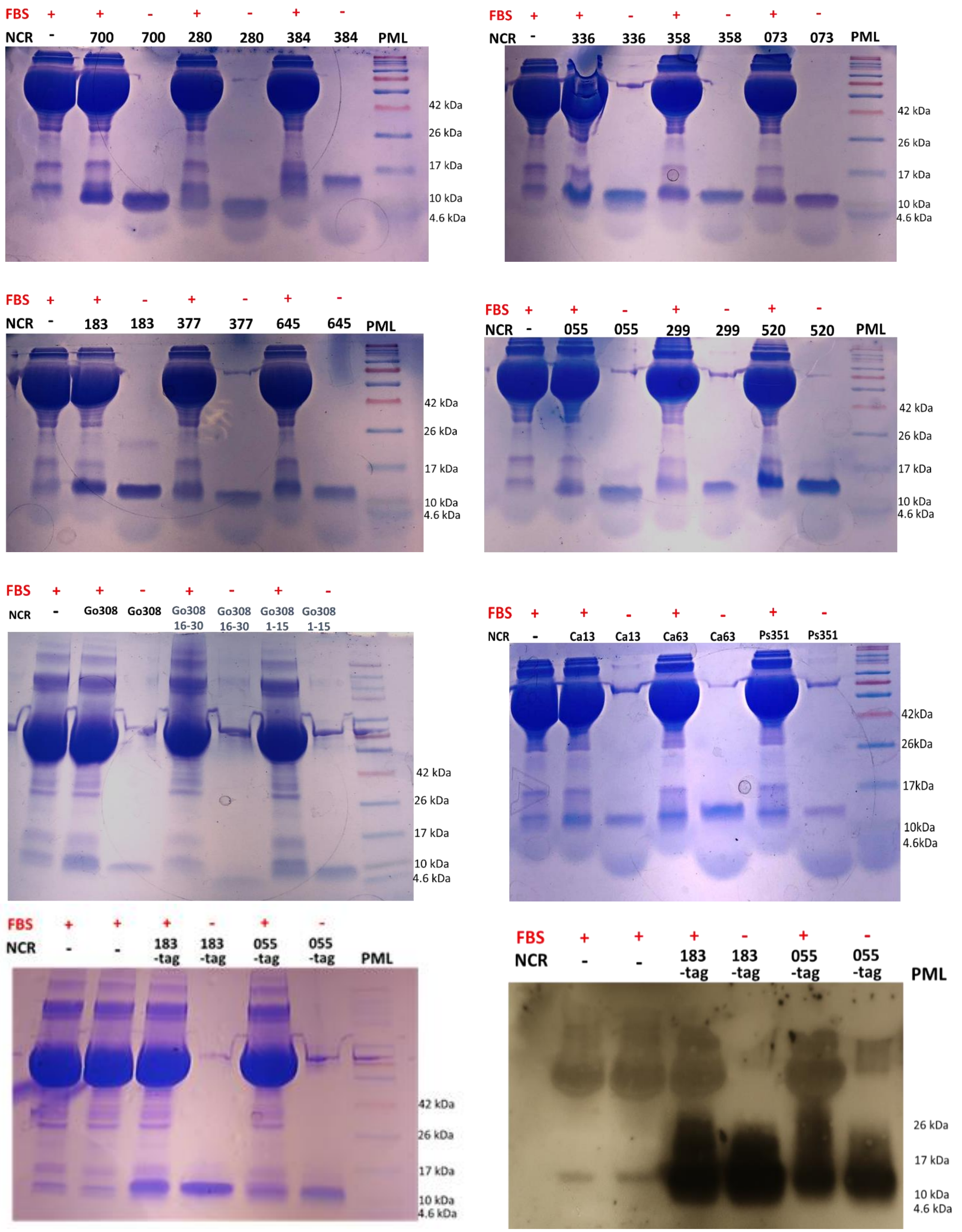

Supplement: Supplementary file 3 [file DataSheet1.PDF]
